# Digital spatial profiling identifies the tumor center as a topological niche in prostate cancer characterized by an upregulation of BAD

**DOI:** 10.1038/s41598-024-71070-6

**Published:** 2024-08-31

**Authors:** Ann-Kathrin Huber, Adam Kaczorowski, Felix Schneider, Sarah Böning, Magdalena Görtz, David Langhoff, Constantin Schwab, Albrecht Stenzinger, Markus Hohenfellner, Anette Duensing, Stefan Duensing

**Affiliations:** 1 Molecular Urooncology, Department of Urology, University Hospital Heidelberg, Im Neuenheimer Feld 517, 69120 Heidelberg, Germany; 2 Department of Urology, University Hospital Heidelberg, and National Center for Tumor Diseases (NCT), Im Neuenheimer Feld 420, 69120 Heidelberg, Germany; 3 Institute of Pathology, University Hospital Heidelberg, Im Neuenheimer Feld 224, 69120 Heidelberg, Germany; 4 Precision Oncology of Urological Malignancies, Department of Urology, University Hospital Heidelberg, Im Neuenheimer Feld 517, 69120 Heidelberg, Germany

**Keywords:** Prostate cancer, Spatial biology, Intratumoral heterogeneity, Tumor center, BAD, Cancer, Tumour heterogeneity, Urological cancer

## Abstract

Prostate cancer is characterized by a high degree of intratumoral heterogeneity. However, little is known about the spatial distribution of cancer cells with respect to specific functional characteristics and the formation of spatial niches. Here, we used digital spatial profiling (DSP) to investigate differences in protein expression in the tumor center *versus* the tumor periphery. Thirty-seven regions of interest were analyzed for the expression of 47 proteins, which included components of the PI3K-AKT, MAPK, and cell death signaling pathways as well as immune cell markers. A total of 1739 data points were collected from five patients. DSP identified the BCL-2 associated agonist of cell death (BAD) protein as the most significantly upregulated protein in the tumor center. BAD upregulation was confirmed by conventional immunohistochemistry, which furthermore showed a phosphorylation of BAD at serine 112 indicating its inactivation. Knockdown of BAD in prostate cancer cells in vitro led to decreased cell viability and colony growth. Clinically, high BAD expression was associated with a shorter time to biochemical recurrence in 158 mostly high-risk prostate cancer patients. Collectively, our results suggest that the tumor center is a topological niche with high BAD expression that may drive prostate cancer progression.

## Introduction

Prostate cancer is the most common non-cutaneous cancer and the third leading cause of cancer death in Western men^1^. It shows considerable variability in aggressiveness and the propensity for disease progression, ranging from indolent to lethal disease^2^. Prostate cancer is characterized by a high degree of intratumoral heterogeneity. Intratumoral heterogeneity comprises genomic as well as functional heterogeneity and is thought to represent a major cause for treatment failure and ultimately lethal disease outcome^3–5^.

The prostate cancer “ecosystem” not only consists of a heterogenous population of tumor cells but also an equally heterogenous population of non-malignant cells such as immune cells, fibroblasts, and others^4,6,7^. In this context, it appears conceivable that topological niches exist that are populated by tumor cells with certain functional characteristics. The most obvious spatial niches are the tumor center and the tumor periphery since factors such as oxygen supply, pH, and the possibility to interact with heterotopic cell populations are not equally distributed within the tumor^8^. To underscore this notion, it has recently been shown that the tumor periphery in prostate cancer frequently harbors senescent cells, which are permanently withdrawn from the cell division cycle^9^. The concept of intratumoral niches is only beginning to emerge and additional studies are needed.

An emerging method to study spatial biology is the Nanostring GeoMx® digital spatial profiling (DSP) platform. DSP allows a high-plexed analysis of protein or mRNA expression in spatially defined regions of interest (ROIs) using antibodies or mRNA probes labeled with UV-cleavable oligonucleotide barcodes. After selective UV exposure, the photocleavable barcodes are released and counted to provide a spatially resolved profile of protein or mRNA abundance. ROI selection is typically based on morphological markers and tissue landmarks such as tumor center and tumor periphery can be readily analyzed^4,10,11^.

Whether and to what extent spatial niches are involved in tumor progression is largely unknown. Tumor progression ultimately requires an activation of anti-apoptotic and pro-survival pathways. For example, it has been shown that anti-apoptotic proteins of the BCL-2 family are involved in driving prostate cancer progression^12,13^. A recent study in renal cell carcinoma reported more aggressive subclonal growth characteristics in the tumor center, suggesting that the tumor center may play a critical role in tumor progression and metastatic dissemination^14^.

In the present proof-of-concept study, we used DSP of protein expression to analyze the tumor center and the tumor periphery in prostate cancer. We identified the tumor center as a spatial niche characterized by the upregulation of the BCL-2 agonist of cell death (BAD) protein and show that overexpression of BAD has a negative impact on disease progression and patient prognosis.

## Results

### DSP of protein expression in the tumor center and periphery in primary prostate cancer

To interrogate the tumor center and the tumor periphery as spatial niches in prostate cancer, we used the NanoString GeoMx® DSP platform^10^.

**Fig. 1:**
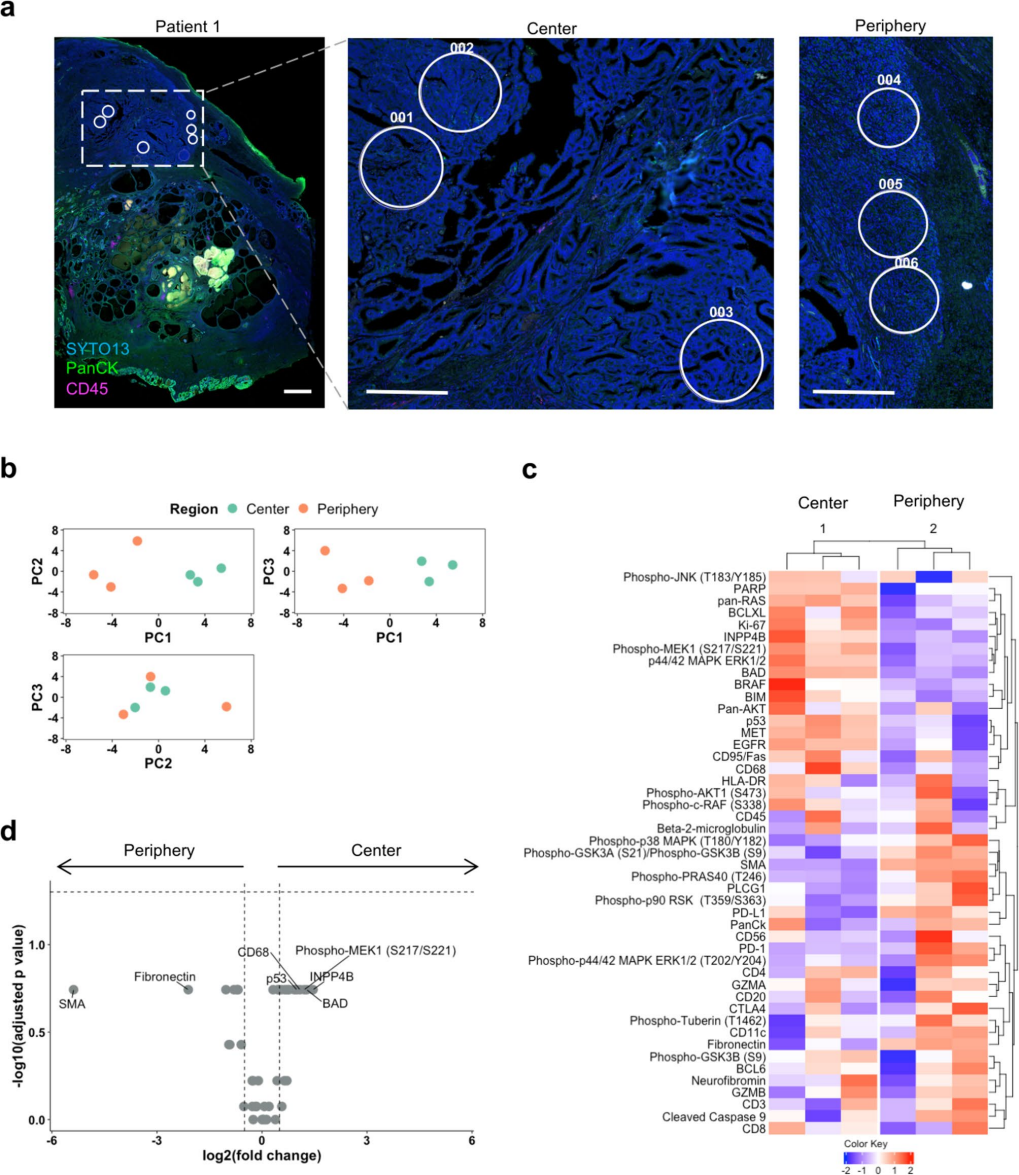
Digital spatial profiling (DSP) of protein expression to compare tumor center and tumor periphery in prostate cancer. (**a**) Regions of interest (ROIs) selected to represent the tumor center and periphery in patient 1. Tissue was stained with anti-PanCK (green), anti-CD45 (pink), and SYTO13 (blue, nuclear staining) for visualization. All ROIs were diagnosed as Gleason grade 4. Scale bars = 1 mm (left panel); 500 µm (middle and right panel). (**b**) Principal component analysis (PCA) of ROIs from the tumor center and periphery. (**c**) Unsupervised clustering and heatmap of DSP protein expression data obtained from six ROIs. For clustering, protein expression values were z-transformed by subtracting the protein mean from each protein and dividing by its standard deviation. Z-scores are presented according to the color key. (**d**) Volcano plot showing differentially expressed proteins between the tumor center and periphery. The vertical dotted lines represent the log_2_(fold change) of 0.5 and − 0.5. The horizontal dotted line shows the *p*-value cut-off of 0.05. *P*-values were determined by Mann–Whitney U test.

In a pilot experiment, we selected six ROIs, three from the tumor center and three from the tumor periphery, to investigate protein expression (Patient 1; Fig. 1a). Principal component analysis and unsupervised hierarchical clustering showed a clear separation of tumor center and periphery (Fig. 1b, c).

A number of up- and downregulated proteins were detected in the tumor center and the tumor periphery (Fig. 1d). Proteins upregulated in the tumor center included proteins involved in apoptosis regulation (p53 and BAD), INPP4B as a component of the PI3K/AKT signaling network, phospho-MEK1 (S217/S221) as a component of the MAPK signaling network and the monocyte/macrophage marker CD68. In the tumor periphery, the most differentially expressed proteins were proteins involved in epithelial-to-mesenchymal transition (SMA, fibronectin).

Although these results did not reach statistical significance (Fig. 1d), they encouraged us to include four additional tumors in the analysis.

### DSP identifies the tumor center as an intratumoral niche with BAD upregulation

**Fig. 2:**
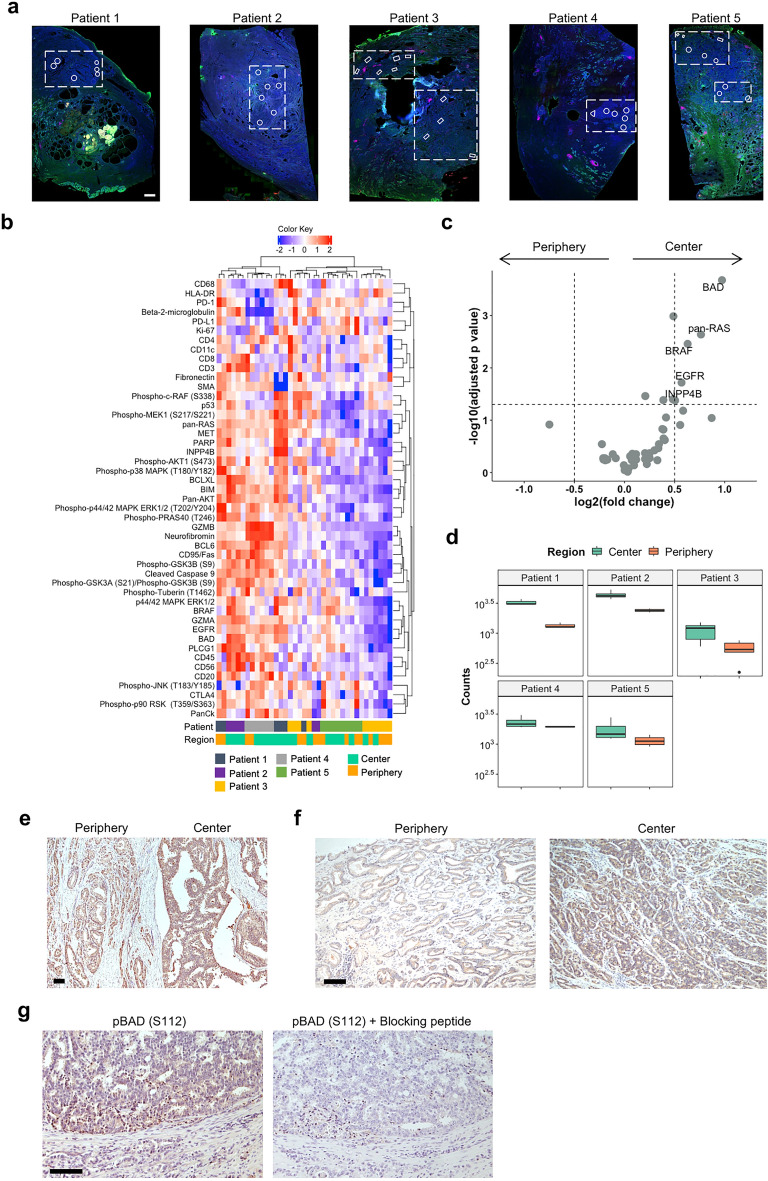
DSP identifies the tumor center as an intratumoral niche with high BAD expression. (**a**) Stained tumor sections from all five patients subjected to pooled DSP profiling analysis comparing tumor center and periphery. Tissue was stained with anti-PanCK (green), anti-CD45 (pink), and SYTO13 (blue, nuclear staining) for visualization. Scale bar = 1 mm. (**b**) Unsupervised clustering and heatmap of DSP protein expression data obtained from five patients and 37 ROIs. For clustering, protein expression values were z-transformed by subtracting the protein mean from each protein and dividing by its standard deviation. Z-scores are presented according to the color key. (**c**) Volcano plot depicting differentially expressed proteins between tumor center and periphery. The vertical dotted lines represent the log_2_(fold change) of 0.5 and − 0.5. The horizontal dotted line shows the *p*-value cut-off of 0.05. P-values were determined using a linear mixed model (LMM) after log_2_-transformation of the data. (**d**) Box plots of BAD expression counts in the tumor center and the tumor periphery of each patient. (**e–g**) Representative tumor tissue from DSP patient 1 stained for BAD (**e**), a representative patient outside the DSP cohort stained for BAD (**f**), and phospho-BAD (S112) staining of patient 1 (**g**), respectively. The specificity of the phospho-antibody was determined using a epitope-specific blocking peptide. Scale bars = 100 μm.

We profiled prostate cancer tissue from a total of five patients (Table 1) and selected representative ROIs in the tumor center and the tumor periphery (Fig. 2a). A total of 37 ROIs (21 from the tumor center and 16 from the tumor periphery) were analyzed (Fig. 2b, c).

**Table 1:** Clinico-pathological patient characteristics (DSP).

Patient characteristics (n = 5)	
Age, years, median (range)	65 (51–67)
PSA level at diagnosis, ng/ml, median (range)	9.6 (6.4–27.6)
ISUP grade group, Gleason score, n (%)	
2 (3 + 4)	2 (40)
3 (4 + 3)	0 (0)
4 (8)	1 (20)
5 (9–10)	2 (40)
pT stage, n (%)	
T2	1 (20)
T3	4 (80)
pN stage, n (%)	
N1	2 (40)
Nx	3 (60)
c/pM stage, n (%)	
M1	1 (20)
Mx	4 (80)
R status	
R0	2 (40)
R1	3 (60)

Although the cluster analysis did not yield a clear separation between tumor center and tumor periphery (Fig. 2b), analysis of the five tumors showed a number of proteins that were significantly upregulated (*p* < 0.05 and log_2_(fold change) > 0.5) in the tumor center (Fig. 2c). Among these proteins were BAD, pan-RAS, BRAF, EGFR, and INPP4B. Notably, BAD was the most significantly upregulated protein in the tumor center.

BAD was found to be consistently overexpressed in the tumor center of all five patients (Fig. 2d). For further validation purposes, we also stained all five patient samples for BAD expression using conventional immunohistochemistry. All tumors showed higher BAD expression in the tumor center compared to the tumor periphery, as illustrated for patient 1 (Fig. 2e). Additional immunohistochemical stainings for BAD on 18 prostate cancer samples obtained from 12 patients also showed a higher BAD expression in the tumor center than in the tumor periphery (Fig. 2f) in all but one sample. There was no statistically significant correlation between the ROI localization (center vs. periphery) and the Gleason grade (*p* > 0.05, Fisher's Exact test; Suppl. Table 1).

BAD has anti-apoptotic, pro-survival functions in its phosphorylated state^15^. We found that BAD was phosphorylated at S112 in the tumor center (Fig. 2g).

Taken together, these results suggest the tumor center as an intratumoral niche characterized by an upregulation of phosphorylated and therefore anti-apoptotic BAD.

### BAD expression promotes prostate cancer cell survival and growth

Since we identified BAD as the most significantly upregulated protein in the tumor center, we next investigated whether BAD could drive prostate cancer progression through a series of in vitro experiments.

**Fig. 3:**
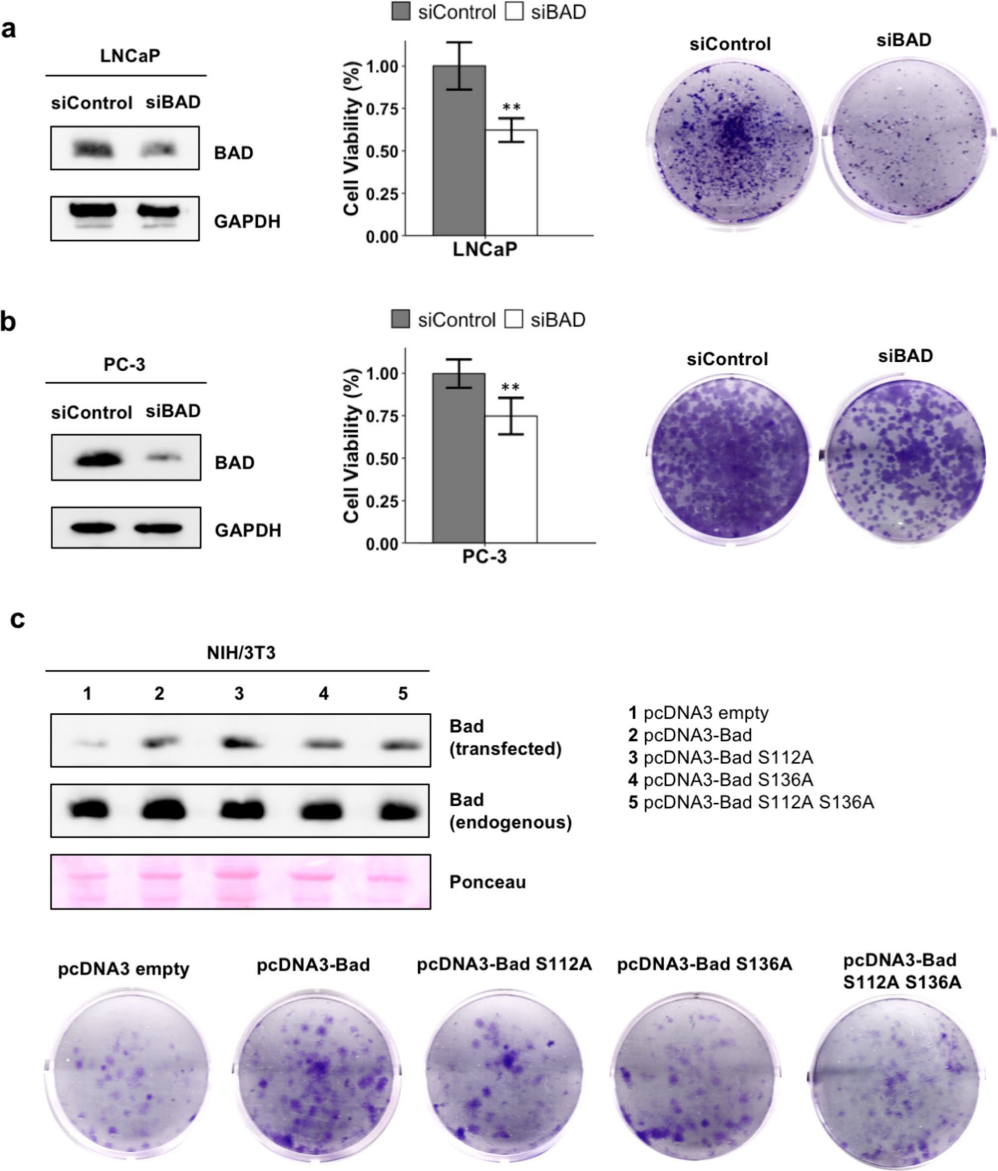
BAD expression affects cell survival and growth. (**a**, **b**) SiRNA-mediated knockdown of BAD in LNCaP (**a**) and PC-3 (**b**) cells. The knockdown was confirmed by immunoblot analysis. GAPDH was used as a loading control (left panels). Cell viability of control and BAD-depleted LNCaP (**a**) and PC-3 (**b**) cells was determined by MTT assay five days after transfection. Experiments were performed in duplicate. A Mann–Whitney U test was used to assess statistically significant differences in cell viability (middle panels; **, *p *< 0.005). Colony-forming ability in siRNA-transfected BAD-depleted LNCaP (**a**) and PC-3 (**b**) cells was assessed by colony formation assays (right panels). Representative images are shown. Experiments were performed in triplicate. (**c**) Bad overexpression in NIH/3T3 cells. After transfection with murine pcDNA3-Bad, phospho-mutants pcDNA3-Bad S112A, pcDNA3-Bad S136A and pcDNA3-Bad S112A S136A or control DNA, overexpression was confirmed by immunoblot analysis. Equal protein loading was demonstrated by Ponceau staining (upper panels). The effects of overexpression of wild-type Bad and Bad mutants on colony formation (lower panels) was evaluated. Representative images are shown. Experiments were performed in triplicate. Original immunoblots and Ponceau staining are shown in Suppl. Fig. S2.

Using small interfering RNA (siRNA), we generated a knockdown of BAD in LNCaP and PC-3 prostate cancer cells. Reduced expression of BAD was confirmed by immunoblot analysis. We found a significant reduction in cell viability in both LNCaP (1.6-fold reduction; *p* = 0.0025) and PC-3 (1.3-fold reduction; *p* = 0.0043) cells. At the same time, siRNA-mediated BAD knockdown reduced colony growth in both LNCaP and PC-3 cells (Fig. 3a, b).

To investigate whether overexpression of BAD had the opposite effect, we transfected NIH/3T3 cells with a plasmid expressing murine Bad as well as a series of Bad phospho-mutants (Bad S112A, Bad S136A and Bad S112A S136A). Overexpression was confirmed by immunoblot analysis. We detected enhanced colony growth in Bad-transfected NIH/3T3 cells (Fig. 3c). Overexpression of the phospho-mutants caused a moderate but clearly discernible reduction of colony growth when compared to the overexpression of wild-type Bad. Hence, the phosphorylation of Bad is required to promote colony growth.

Taken together, these results indicate that BAD expression supports cell survival and growth of prostate cancer cells in vitro.

### High BAD expression correlates with a shorter time to biochemical recurrence

**Fig. 4:**
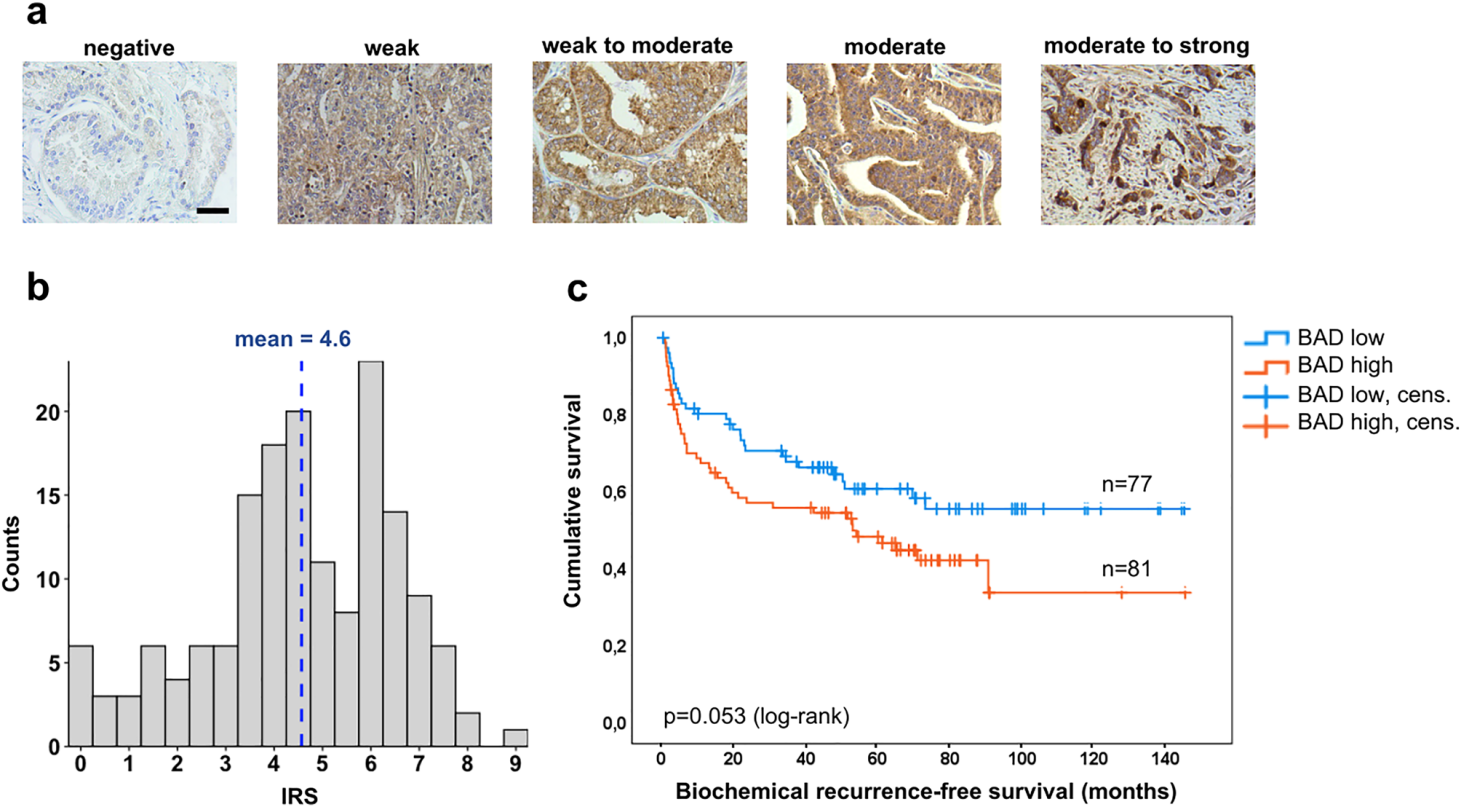
BAD expression has prognostic relevance in prostate cancer. (**a**) Representative immunohistochemical stainings showing different expression levels of BAD. Scale bar = 50 µm. (**b**) Histogram showing the distribution of BAD immunoreactive scores (IRS) per patient. (**c**) Kaplan–Meier curve for 158 patients, stratified into patients with high (> 4.6; n = 81) or low (≤ 4.6; n = 77) BAD IRS. Cens., censored.

To further analyze the role of BAD in prostate cancer progression, we first analyzed a cohort of 24 patients, for which corresponding specimens consisting of non-tumorous tissue (n = 45), prostate cancer Gleason grade 3 (n = 45) and grade 4 (n = 45) were assembled into a tissue microarray (TMA) with a total of 135 cores. A BAD immunoreactivity score (IRS) was calculated by multiplying the signal intensity of the BAD staining by the percentage of positive cells. The signal intensity was scored on a scale of 0 to 2.5, with 0 = negative, 1 = weak, 1.5 = weak to moderate, 2 = moderate and 2.5 = moderate to strong (Fig. 4a). The percentage of positive cells was defined as follows: negative staining was scored as 0, < 10% of stained cells was scored as 1, 10–50% was scored as 2, 50% to 80% was scored as 3, and > 80% was scored as 4.

Although the tumor center was not enriched for a higher Gleason grade (see above), there was a statistically significant increase of BAD expression between benign tissue and Gleason grade 3 and grade 4 prostate cancer. The mean BAD IRS was 5.2 in non-tumorous tissue, 5.5 in Gleason grade 3 prostate cancer (*p* = 0.008) and 6.6 in Gleason grade 4 prostate cancer (*p* = 0.00002, Mann-Whitney U test; Suppl. Fig. S1).

To ascertain whether an upregulation of BAD plays a role in patient prognosis, a TMA analysis of 158 prostate cancer specimens from mostly high-risk patients was performed (Table 2). Most patients in the study cohort had ISUP grade 4 or 5, pT stage 3 and positive lymph nodes. Biochemical recurrence occurred in 47% of patients.

**Table 2:** Clinico-pathological patient characteristics (TMA).

Patient characteristics (n = 158)	
Age, years, median (range)	66 (48–79)
Biochemical recurrence, n (%)	
Yes	74 (47)
No	84 (53)
ISUP grade group, Gleason score, n (%)	
2 (3 + 4)	20 (13)
3 (4 + 3)	21 (13)
4 (8)	51 (32)
5 (9–10)	65 (41)
Missing information	1(1)
pT stage, n (%)	
T2	25 (16)
T3	118 (75)
T4	15 (9)
pN stage, n (%)	
N0	43 (27)
N1	115 (73)
c/pM stage, n (%)	
M0	153 (97)
M1	5 (3)
R status	
R0	49 (31)
R1	70 (44)
Rx	39 (25)

For each of the 526 TMA cores, the BAD IRS was calculated as described above.

A mean of 3.3 cores were evaluated for each patient. The mean IRS of all evaluable TMA cores per patient was considered the IRS of an individual patient. The BAD IRS distribution ranged from 0 to 9 with a mean of 4.6 (Fig. 4b). When patients were stratified into two groups according to BAD IRS, Kaplan–Meier analysis showed that patients with high BAD expression (BAD IRS > 4.6) tended to have a shorter time to biochemical recurrence (*p* = 0.053; Fig. 4c).

Taken together, these results suggest that an upregulation of BAD is associated with more aggressive histopathological features and a more unfavorable progression-free survival of prostate cancer patients.

## Discussion

Prostate cancer is characterized by a high degree of intratumoral heterogeneity^3–5^. Several studies have shown that intratumoral heterogeneity involves the formation of spatial niches, i.e., tumor areas that are populated by tumor cells with certain functional characteristics^9,16–19^. The most obvious intratumoral niches are the tumor center and the tumor periphery based on differences in oxygen supply, pH, and the extent of heterotopic cellular interactions^8^. The tumor periphery in renal cell carcinoma has previously been reported to show an enrichment of proliferating cells^16,20^. In stark contrast, signs of cellular senescence have been observed in the tumor periphery of thyroid cancer^19^ and prostate cancer^9^. Whether the tumor center also represents a spatial niche harboring cells with certain functional properties is incompletely understood.

In the present study, we used DSP to investigate protein expression in the tumor center and the tumor periphery of five radical prostatectomy specimens. DSP identified the BCL-2-family protein BAD as the most significantly upregulated protein in the tumor center. Validation experiments showed that upregulated BAD in the tumor center was phosphorylated and therefore inactive as pro-apoptotic factor. In vitro experiments demonstrated a role for BAD in tumor progression as overexpression enhanced cell viability and colony formation. Higher BAD expression also correlated with shorter time to biochemical recurrence in a cohort of high-risk prostate cancer patients. These findings underscore the role of the tumor center in prostate cancer as a spatial niche characterized by BAD upregulation that may contribute to tumor progression and may also influence patient prognosis.

To the best of our knowledge (PubMed search terms: prostate cancer, tumor center, niche; search date: 10th July 2024), this is the first study to provide experimental evidence that the tumor center may be a spatial niche with relevance for tumor progression and thus strongly supports a model of intratumoral niche formation in prostate cancer.

It has previously been observed that prostate cancer has an increased BAD expression compared to healthy prostate tissue^21^. High-grade tumors (Gleason Score > 6) were shown to express more BAD than low-grade tumors^22^. Similar findings have been described for other tumor entities when compared to their respective non-malignant counterpart^23^. The present study confirms and extends these results by showing a significant increase of BAD expression from non-tumorous tissue to Gleason grade 3 and 4 prostate cancer in matching patient samples.

An important finding of our analysis is that BAD is predominantly phosphorylated in the tumor center. BAD is a pro-apoptotic protein that belongs to the BCL-2 family. It localizes to the mitochondria and is involved in the mitochondrial pathway of apoptosis. However, the pro-apoptotic effect of BAD is disabled by phosphorylation. In the phosphorylated state, BAD is sequestered in the cytosol and acts as a pro-survival factor^15^. Increased expression of phosphorylated BAD is associated with tumor progression and resistance to therapy in a variety of cancers, including breast, ovarian, endometrial, gastric and liver cancer as well as malignant melanoma^23^. In line with this, our in vitro experiments also show that prostate cancer cells rely on BAD for cell viability and colony formation. Our results are consistent with a previous study by Smith et al. that showed increased cell growth in vitro and in vivo after the overexpression of BAD^24^. BAD phosphorylation is also required for the survival of prostate cancer stem cells^22^.

There are a number of mechanisms by which BAD could promote tumor progression. Sequestration of anti-apoptotic, phosphorylated BAD in the cytosol has been shown to attenuate pro-apoptotic signaling pathways^15^. Furthermore, it has been shown that survival factor-mediated BAD phosphorylation raises the mitochondrial threshold to release cytochrome c to induce apoptosis^25^. In addition to its major role in the intrinsic apoptosis pathway, functions of phosphorylated BAD in glucose metabolism, cell cycle progression and autophagy regulation have been described, which may further contribute to malignant progression^15,23^.

Consistent with its function in prostate cancer viability and growth, we also provide evidence that BAD may be a negative prognostic factor in men with prostate cancer, as patients with higher expression of BAD tended to have a shorter time to biochemical recurrence. BAD has also been shown to have prognostic relevance in breast cancer, as aggressive triple-negative breast cancers are enriched for BAD expression^26^. In glioblastoma, a high BAD phosphorylation was associated with a shorter overall survival^27^. Interestingly, a previous study found that in androgen-dependent prostate cancer, high BAD expression at diagnosis was associated with longer time to biochemical recurrence^28^. One explanation for this contrasting finding may be the fact that patients in this study received androgen-deprivation therapy (ADT) as part of the study protocol. It is known that ADT alters the expression and function of members of apoptotic pathways^29^. Enzalutamide, a novel antihormonal agent used to treat advanced prostate cancer, has been shown to lead to an activation of PI3K/AKT and BAD phosphorylation thereby potentially causing drug resistance^30^. BAD has also been implicated in castration-resistant prostate cancer, which is androgen-independent. These tumors frequently show a loss of *PTEN* and an activation of the PI3K/AKT and/or RAS/RAF/MEK/ERK pathway^31^. All these events can lead to BAD phosphorylation and its subsequent inactivation by complex formation with 14–3–3^15^ thus promoting tumor cell survival.

What exactly causes BAD upregulation in the tumor center remains to be determined. Hypoxia and pH changes could be a possible cause of BAD overexpression. Previously, BCL-X_L_, another anti-apoptotic member of the BCL-2 family besides BAD, has been shown to be transcriptionally upregulated by hypoxia-inducible factor 1α^32^. In the glioblastoma cell lines U87MG and A172GBM, it was found that hypoxia induces the phosphorylation of BAD at S112^33^. In addition, senescent cells, which are frequently detected in the tumor periphery in prostate cancer^9^, may have an influence on the expression and function of BAD in the tumor center, since senescent cells are known to secrete cytokines and growth factors as part of the senescence-associated secretory phenotype, which may cause an activation of kinases that can phosphorylate BAD^34^.

A study by Zhao et al. has used mathematical modeling to show that the tumor center in renal cell carcinoma is a site of more aggressive clonal growth and thus represents a niche of central importance for the development of metastatic subclones^14^. Although this concept remains to be proven experimentally, it may lend support to our notion that the tumor center may represent a spatial niche with relevance for tumor progression.

There are currently a number of techniques available to interrogate tumor heterogeneity including single cell RNA sequencing (scRNA-seq) and spatial transcriptome or proteome analysis (the latter used herein). While scRNA-seq is an excellent tool to analyze cellular heterogeneity, cells have to be separated before the actual analysis. Spatial information hence needs to be gathered immediately before or at the time of sampling with thus leaving some degree of uncertainty with respect to the precise cellular localization. Although scRNA-seq allows the analysis of the entire transcriptome, one caveat is that changes in mRNA expression may not necessarily translate into the same degree of change on the protein level, a notion that also applies to sequencing-based spatial transcriptomics^35^. DSP is a spatial multi-omics approach that relies on barcoded hybridization probes and therefore does not require tissue disaggregation^36^. Moreover, DSP is well-suited for archived formalin-fixed, paraffin-embedded tissue^10^ whereas scRNA-seq usually requires fresh tissue samples. While single cell proteomics is still an emerging technique^37^, spatial protein expression profiling has become more widely available in the past several years^36^. It needs to be emphasized, though, that one disadvantage lies in the fact that the number of simultaneously detected proteins is typically limited and results also depend on the quality of the antibodies used^10^. On the other hand, the functional state of cells can be deduced using phospho-specific antibodies. In the method used in the present paper, no single cell annotation is possible but there are newer systems with single-cell resolution^38^.

Limitations of this study are the small sample size and the lack of direct mechanistic proof of how precisely BAD contributes to tumor progression. While tumor center and periphery are two obvious intratumoral compartments that may differ in a number of parameters such as oxygen supply or pH^39^, they may not fully represent the entirety of factors that drive malignant progression. Other intratumoral niches may for example, be represented by the perivascular space or by areas adjacent to necrotic tissue^14,40^. In addition, the tumor microenvironment is very likely to contribute to tumor progression^41^ but has not been included in the present analysis. Moreover, while DSP provides spatially annotated gene expression data in a two-dimensional fashion, it needs to be emphasized that three-dimensional analyses may provide additional insights into the architecture of a tumor, in particular prostate cancer, which is known to present with a highly scattered distribution of tumor cells^3^.

The translational relevance of our study may be that targeting BAD could be used to inhibit tumor progression or tumor clones with the ability to survive and to promote tumor progression^23^.

## Conclusion

The results of this proof-of-concept study suggest that the tumor center may function as a topological niche in prostate cancer with implications for tumor progression and patient risk stratification.

## Patients and methods

### Patients

Formalin-fixed, paraffin-embedded (FFPE) tissue sections from five patients with primary, treatment-naive prostate cancer were analyzed using the GeoMx® DSP platform (NanoString Technologies, Seattle, WA, USA) (Table 1). All patients underwent radical prostatectomy at the Department of Urology, University Hospital of Heidelberg, Germany, between 2007 and 2021.

BAD expression was evaluated in a TMA cohort consisting of prostate cancer specimens from 158 patients who underwent radical prostatectomy at the Department of Urology, University Hospital of Heidelberg, Germany, between 1990 and 2012 (Table 2). In addition, a TMA consisting of 135 cores from 24 prostate cancer patients with matching non-tumorous tissue and prostate cancer Gleason grade 3 and 4 was analyzed.

All tissue sections were provided by the tissue bank of the National Center for Tumor Diseases (NCT) Heidelberg. All experiments were approved by the tissue bank and the Ethics Committee of the Medical Faculty Heidelberg of the University of Heidelberg (S-206/2005, S-207/2005, S-864/2019, S-130/2021).

### Digital spatial profiling of protein expression

FFPE sections were deparaffinized in xylol and rehydrated in ethanol (100% and 95%) and ddH_2_O. Antigen retrieval was performed in a pressure cooker using 1X citrate buffer (pH 6.0). Tissue sections were blocked with Buffer W (NanoString) for 1 h at room temperature. After blocking, the slides were incubated with barcoded antibody panels (NanoString) overnight at 4 °C in a humidity chamber. The barcoded antibody modules used were the GeoMx® Human Protein Immune Cell Profiling module (24 protein targets including negative controls and housekeeping genes), the GeoMx® PI3K/AKT (9 protein targets) and MAPK (10 protein targets) Signaling modules, and the GeoMx® Cell Death module (10 protein targets) (Table 3). To visualize tissue architecture, the slides were stained for pan-cytokeratin (PanCK; epithelial/tumor cells) and CD45 (immune cells) using the Solid Tumor TME Morphology Kit (NanoString). After antibody staining, slides were postfixed in 4% paraformaldehyde at room temperature and nuclei were stained with SYTO13 (NanoString). Slides were scanned with the GeoMx® instrument (v.2.4.2.2) and ROIs were selected in the tumor center and tumor periphery by a trained pathologist (S.D.). To aid in ROI selection, consecutive hematoxylin- and eosin-stained sections were simultaneously examined by light microscopy. Each ROI was illuminated with UV light and cleaved barcodes were collected and hybridized with fluorescent probes (16 h at 67 °C). Hybridized probes were processed using the nCounter® MAX/FLEX Prep Station (v4.1.0.1, NanoString) and counted using the nCounter® Digital Analyzer (v4.0.0.3, NanoString). Quality control was performed to remove low-quality samples from the profiling analysis, and counts were normalized to the geometric mean of the negative controls (mouse IgG1, IgG2a). Visualization of the data was achieved using the GeoMx® DSP analysis suite and R (v.4.2.0).

**Table 3:** Overview of NanoString GeoMx® human protein assays.

Immune cell profiling	PI3K/AKT signaling	MAPK signaling	Cell death
Beta-2-microglobulin	Phospho-AKT1 (S473)	EGFR	BAD
CD11c	Phospho-GSK3B (S9)	Pan-RAS	BCL6
CD20	Phospho-GSK3A (S21)/Phospho-GSK3B (S9)	BRAF	BCLXL
CD3	INPP4B	Phospho-c-RAF (S338)	BIM
CD4	PLCG1	Phospho-JNK (T183/Y185)	CD95/Fas
CD45	Phospho-PRAS40 (T246)	Phospho-MEK1 (S217/S221)	GZMA
CD56	Phospho-Tuberin (T1462)	Phospho-p38 MAPK (T180/Y182)	p53
CD68	Pan-AKT	Phospho-p44/42 MAPK ERK1/2 (T202/Y204)	PARP
CD8	MET	p44/42 MAPK ERK1/2	Cleaved Caspase 9
CTLA4		Phospho-p90 RSK (T359/S363)	Neurofibromin
PanCK			
Fibronectin			
GZMB			
HLA-DR			
Ki-67			
PD-1			
PD-L1			
SMA			
Ms IgG2a			
Ms IgG1			
Rb IgG			
Histone H3			
S6			
GAPDH			

### Immunohistochemistry

FFPE tissue sections were deparaffinized in xylol and rehydrated in a graded ethanol series (100%, 90%, and 70%). Heat-mediated antigen retrieval was performed using antigen retrieval solution (Dako, Glostrup, Denmark) and slides were blocked in goat serum for 1 h. Anti-Bad (Y208, ab32445, 1:200, Abcam, Cambridge, UK) and anti-phospho-Bad (Ser112) (40A9, #5284, 1:50, Cell Signaling Technology, Danvers, MA, USA) were used as primary antibodies. Primary antibody detection was performed with biotinylated anti-rabbit secondary antibody (ab97049, 1:200; Abcam) and streptavidin-peroxidase conjugate (1:1250, Sigma-Aldrich, Taufkirchen, Germany). 3,3′-Diaminobenzidine (DAB) was used as a substrate for the chromogen reaction. Tissue sections were counterstained with Hematoxylin Gill I (Sigma-Aldrich, St. Louis, MO, USA), dehydrated, and mounted with HistoMount solution (Life Technologies, Carlsbad, CA, USA).

Both TMAs were stained for BAD expression according to the described immunohistochemistry protocol. BAD expression was assessed by a semiquantitative IRS considering signal intensity and percentage of positive cells. All samples were reviewed by two independent observers (A.H. and S.D.).

### Cell lines and transfection

The LNCaP and PC-3 human prostate cancer cell lines were purchased from CLS (Eppelheim, Germany), and the NIH/3T3 mouse fibroblast cell line (CRL-1658) was purchased from ATCC (Manassas, VA, USA). LNCaP and PC-3 cell lines were cultured in RPMI 1640 and Ham’s F-12 K medium, respectively, both supplemented with 10% fetal bovine serum. The NIH/3T3 cell line was cultured in DMEM medium supplemented with 10% fetal calf serum. All media were further supplemented with 50 units/ml penicillin, 50 µg/ml streptomycin and 0.5 µg/ml amphotericin B. Cells were grown in a humidified incubator at 37 °C and 5% CO_2_.

For siRNA-mediated BAD knockdown, LNCaP and PC-3 cells were seeded at 0.5–1.0 × 10^5^ cells/well in a 6-well plate. Transfection was performed the next day with either 75 pmol control siRNA (Dharmacon, Chalfont St. Giles, UK) or 75 pmol BAD siRNA (ON-TARGET plus Human Bad siRNA SMART pool, Dharmacon) per well using the DharmaFECT 3 Transfection Reagent (Horizon Discovery, Waterbeach, UK).

For BAD overexpression experiments, 1 × 10^6^ NIH/3T3 cells were transfected with 1 μg of either pcDNA3 control plasmid (#128034, Addgene, Watertown, MA, USA), murine pcDNA3-Bad plasmid (#8778, Addgene) or phospho-mutants pcDNA3-Bad S112A (#8797, Addgene), pcDNA3-Bad S136A (#8798, Addgene) and pcDNA3-Bad S112A S136A (#8799, Addgene) using the Neon Transfection System (Life Technologies). Successful transfection was confirmed by immunoblot analysis.

### Immunoblot analysis

Cell lysis was performed in lysis buffer (50 mM Tris–HCl, pH 8.0) containing 1% NP-40 and protease inhibitor (Roche). The Bradford assay (Bio-Rad Laboratories, Hercules, CA, USA) was used to measure protein concentration. Proteins were separated by SDS-PAGE and blotted onto nitrocellulose membranes (Cytiva, Amersham, UK). Membrane blocking was performed with 5% milk in PBS-T for 1 h. Primary antibody incubation was performed overnight at 4 °C.

Rabbit anti-BAD monoclonal antibody (Y208, ab32445, 1:1000, Abcam) and mouse anti-GAPDH monoclonal antibody (0411, sc-47724, 1:500, Santa Cruz Biotechnology, Dallas, TX, USA) were used as primary antibodies. For detection, the membranes were incubated with horseradish peroxidase (HRP)-conjugated anti-rabbit or anti-mouse secondary antibodies (Invitrogen, Carlsbad, USA), followed by chemiluminescence-mediated visualization.

### Cell viability

The CyQuant MTT Cell Viability Assay Kit (Thermo Fisher Scientific, Waltham, MA, USA) was used to assess cell viability. 48 h after siRNA transfection, 2 × 10^3^ LNCaP or PC-3 cells were seeded in triplicate in 96-well plates and grown for three days. MTT solution was added according to the manufacturer’s protocol. Absorbance was measured at 570 nm. Results were normalized to the siRNA control.

### Colony formation assay

48 h after transfection, cells were trypsinized, resuspended and seeded into 6-well plates (LNCaP 5000 cells/well; PC-3 2000 cells/well; NIH/3T3 1000 cells/well). Colony formation was assessed after 7 and 10 days of growth. Cells were fixed in 4% paraformaldehyde for 10 min at room temperature, stained with 0.1% crystal violet for 15 min, and washed twice with ddH_2_O.

### Statistical analysis and R packages

Statistical analysis of spatial protein expression data was performed using the GeoMx® DSP software (v2.4.2.2). The protein expression profiles of the tumor center and the tumor periphery were compared using the Mann–Whitney U test. The correlation between Gleason score and tumor region was assessed using Fisher's Exact test. A linear mixed model (LMM) with patient identifier as a random effect was applied for comparing log_2_-transformed protein expression data across multiple patients. The Benjamini–Hochberg procedure was used to account for multiple hypothesis testing and to control for false discovery rate. Differences in cell viability were calculated using the Mann–Whitney U test. The Kaplan–Meier analysis was performed with the IBM SPSS Statistics software v27. Significance was defined as a *p*-value ≤ 0.05. R packages used for data visualization were tidyverse (1.3.1), ggpubr (0.4.0) and ComplexHeatmap (2.12.0).

### Supplementary Information

Supplementary Information.

## Data Availability

The data presented in this study are available in the article.
