# Supplementary material for: Digital spatial profiling identifies the tumor center as a topological niche in prostate cancer characterized by an upregulation of BAD
Source: Sci Rep. 2024 Aug 31;14:20281. doi: 10.1038/s41598-024-71070-6 (PMC11366015; doi:10.1038/s41598-024-71070-6)
Supplement: Supplementary file 1 — Supplementary Information. [file 41598_2024_71070_MOESM1_ESM.pdf]

SUPPLEMENTARY INFORMATON

Digital spatial profiling identifies the tumor center as a topological niche in prostate cancer characterized by an upregulation of BAD

Ann-Kathrin Huber[1], Adam Kaczorowski[1], Felix Schneider[1], Sarah Böning[1], Magdalena Görtz[2], David Langhoff[1], Constantin Schwab[3], Albrecht Stenzinger[3], Markus Hohenfellner[2], Anette Duensing[4] and Stefan Duensing[1*]

[1]Molecular Urooncology, Department of Urology, University Hospital Heidelberg, Im Neuenheimer Feld 517, D-69120 Heidelberg, Germany
[2] Department of Urology, University Hospital Heidelberg, and National Center for Tumor Diseases (NCT), Im Neuenheimer Feld 420, D-69120 Heidelberg, Germany
[3]Institute of Pathology, University Hospital Heidelberg, Im Neuenheimer Feld 224, D-69120 Heidelberg
[4]Precision Oncology of Urological Malignancies, Department of Urology, University Hospital Heidelberg, Im Neuenheimer Feld 517, D-69120 Heidelberg, Germany

This PDF file includes:

Supplementary Table 1. Overview of spatial and histopathological information for each patient and each ROI.

Supplementary Figure S1. BAD expression in prostate cancer increases with histopathological grade.

Supplementary Figure S2. Original immunoblots and Ponceau staining of Figure 3.

Supplementary Table 1. Overview of spatial and histopathological information for each patient and each ROI.

		Region	Gleason Grade
**Patient 1**			
	ROI 1	Center	4
	ROI 2	Center	4
	ROI 3	Center	4
	ROI 4	Periphery	4
	ROI 5	Periphery	4
	ROI 6	Periphery	4
**Patient 2**			
	ROI 1	Periphery	3
	ROI 2	Periphery	4
	ROI 3	Center	4
	ROI 5	Center	3
	ROI 6	Center	4
	ROI 7	Center	4
**Patient 3**			
	ROI 1	Periphery	3
	ROI 2	Periphery	3
	ROI 3	Periphery	3
	ROI 4	Periphery	3
	ROI 5	Center	3
	ROI 6	Center	4
	ROI 7	Center	4
	ROI 8	Center	4
	ROI 9	Center	3
	ROI 11	Periphery	4
**Patient 4**			
	ROI 1	Periphery	5
	ROI 2	Periphery	4
	ROI 3	Center	5
	ROI 4	Center	5
	ROI 5	Center	5
	ROI 6	Center	5
**Patient 5**			
	ROI 1	Periphery	4
	ROI 2	Periphery	4
	ROI 3	Periphery	4
	ROI 4	Center	4
	ROI 5	Center	4
	ROI 6	Center	4
	ROI 9	Center	3
	ROI 10	Center	3
	ROI 11	Periphery	4

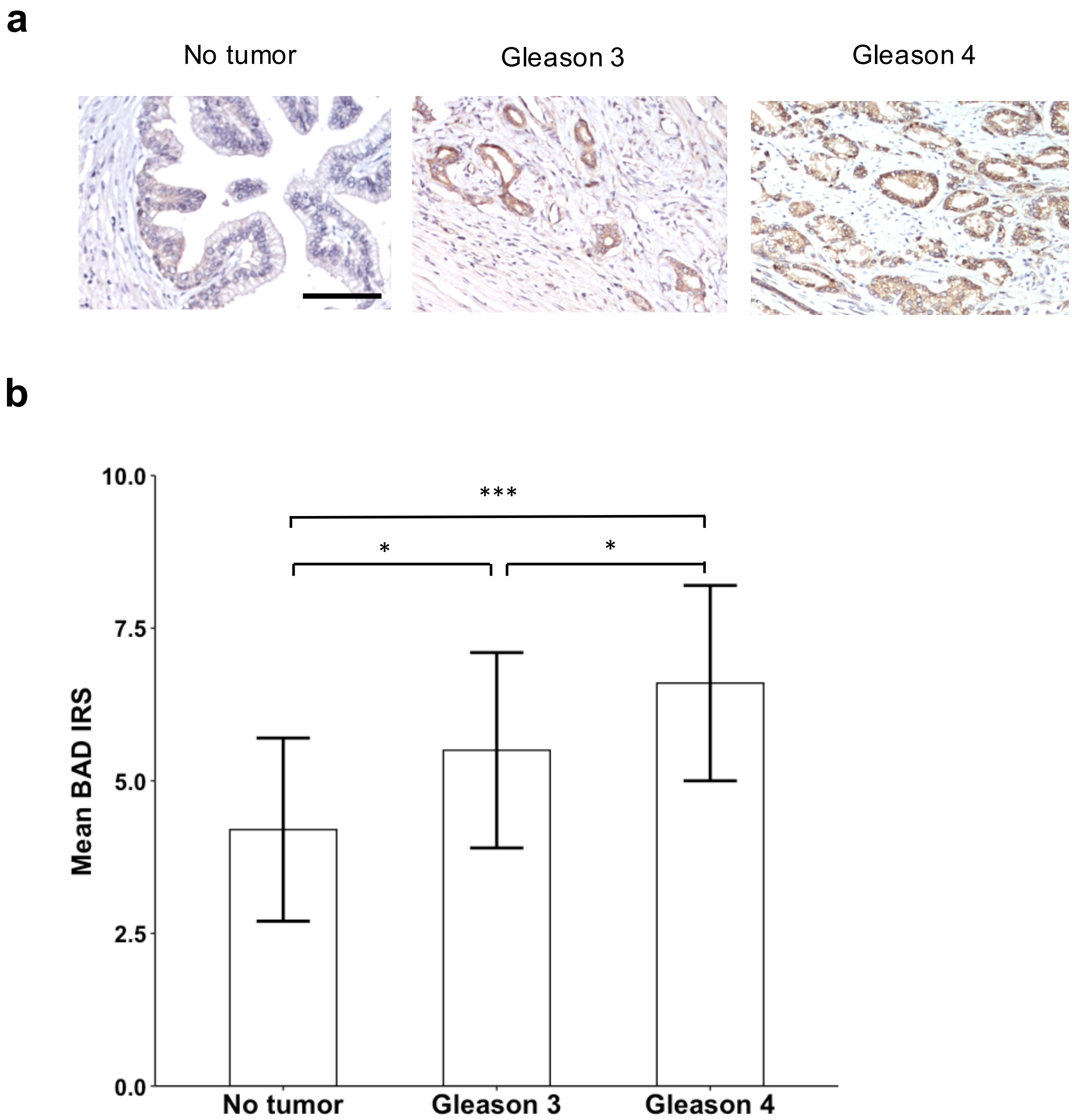

Supplementary Figure S1. BAD expression in prostate cancer increases with histopathological grade. A tissue microarray consisting of 135 cores from 24 prostate cancer patients was stained for BAD. **a)** Representative immunohistochemical stainings showing different levels of BAD expression in non-tumor tissue, Gleason grade 3 and Gleason grade 4 prostate cancer areas from the same patient. Scale bar = 100 µm. **b)** Bar graphs show the mean BAD IRS of non-tumorous tissue (n=45), Gleason grade 3 (n=45) and Gleason grade 4 (n=45) prostate cancer from 24 patients. *, $p<0.05$; ***, $p<0.0005$ (Mann-Whitney U test).

a

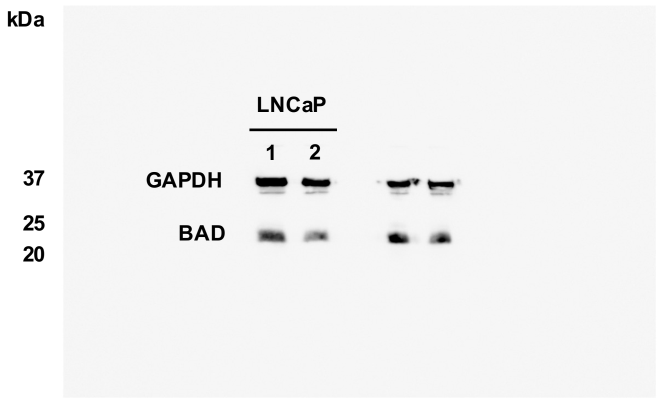

1 siControl
2 siBAD

b

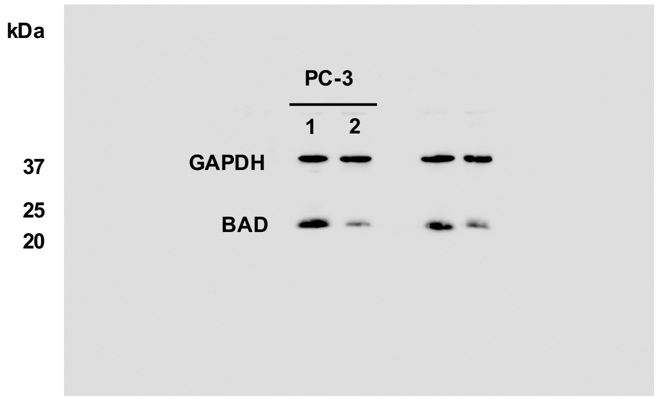

1 siControl
2 siBAD

c

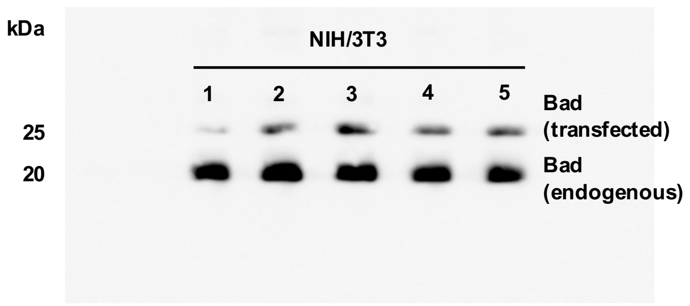

1 pcDNA3 empty
2 pcDNA3-Bad
3 pcDNA3-Bad S112A
4 pcDNA3-Bad S136A
5 pcDNA3-Bad S112A S136A

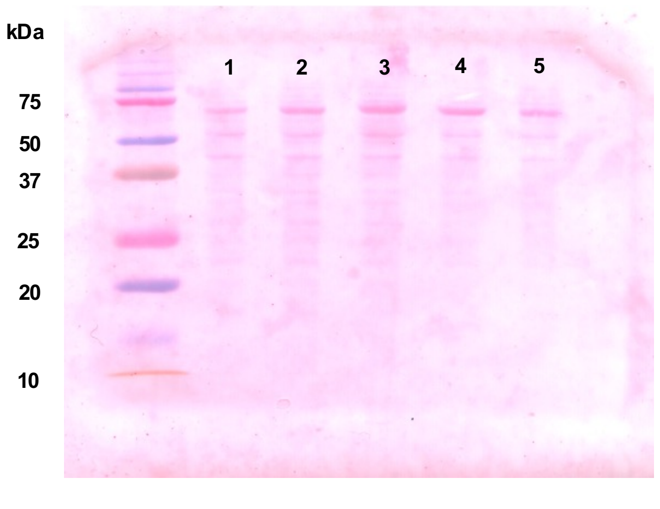

Ponceau

Supplementary Figure S2. Original immunoblots and Ponceau staining of Figure 3. The uncropped images are labelled as shown in Figure 3.
